# Urban Family Planning in Low- and Middle-Income Countries: A Critical Scoping Review

**DOI:** 10.3389/fgwh.2021.749636

**Published:** 2021-10-25

**Authors:** James Duminy, John Cleland, Trudy Harpham, Mark R. Montgomery, Susan Parnell, Ilene S. Speizer

**Affiliations:** ^1^School of Geographical Sciences, University of Bristol, Bristol, United Kingdom; ^2^African Centre for Cities, University of Cape Town, Cape Town, South Africa; ^3^London School of Hygiene and Tropical Medicine, London, United Kingdom; ^4^School of Law and Social Sciences, London South Bank University, London, United Kingdom; ^5^Department of Economics, Stony Brook University, Stony Brook, NY, United States; ^6^Population Council, New York, NY, United States; ^7^Department of Maternal and Child Health, Gillings School of Global Public Health, University of North Carolina, Chapel Hill, NC, United States

**Keywords:** family planning, urban, Africa, Asia, intra-urban differences, critical scoping review

## Abstract

Health agendas for low- and middle-income countries (LMICs) should embrace and afford greater priority to urban family planning to help achieve a number of the global Sustainable Development Goals. The urgency of doing so is heightened by emerging evidence of urban fertility stalls and reversals in some sub-Saharan African contexts as well as the significance of natural increase over migration in driving rapid urban growth. Moreover, there is new evidence from evaluations of large programmatic interventions focused on urban family planning that suggest ways to inform future programmes and policies that are adapted to local contexts. We present the key dimensions and challenges of urban growth in LMICs, offer a critical scoping review of recent research findings on urban family planning and fertility dynamics, and highlight priorities for future research.

## Introduction

Urban family planning (FP) is a pivot point for efforts to achieve the 2030 Sustainable Development Goals (SDGs) because of its centrality to issues of gender, employment, poverty, and health (1). However, like many issues that require cross-sectoral cooperation to make meaningful progress, urban FP has failed to assume the prominence it demands. For the subfield of urban health, for instance, infectious disease, environmental health, and non-communicable diseases are more visible priorities (2). In the context of rapid urban growth and the urbanization of poverty in much of sub-Saharan Africa and South Asia, urban FP should be afforded greater recognition and priority within a broader health agenda.

The urgency of making the case for urban FP is raised by three main factors. Firstly, there is evidence of stalls and reversals in the fertility decline of some African cities, which will potentially sustain rapid urban growth rates in decades to come. Secondly, many urban populations of low- and middle-income countries (LMICs) continue to face high levels of unmet need for contraception, which impacts the socioeconomic trajectories of women. And thirdly, there is now evidence of strategies that can be used to improve effective FP. In particular, the emerging findings of evaluation research on large-scale urban FP programmes—including the Urban Reproductive Health Initiative and its successor The Challenge Initiative—demonstrate the efficacy of well-designed multilevel interventions for increasing the use of FP services among urban populations, including the urban poor. While the efficacy of targeted donor-funded programmes is now established, urgent questions still remain around how best to locate and scale-up FP provision within the routine systems and processes of urban governance.

In critically reviewing the state of knowledge on urban FP in LMIC settings, we emphasize inter-urban and intra-urban dynamics (3). We do not ignore rural-urban comparisons (4–6), and address those differentials where relevant. It is increasingly recognized that an urban-rural dichotomy is insufficient for examining the social, economic, and spatial transformations accompanying contemporary urban growth, including changes related to fertility and health (3, 7, 8). With that in mind, our particular concern is to draw attention to the specific realities and challenges of FP in urban areas, where a growing proportion of LMIC residents live, in order to inform future research, policy, and programme development.

The article proceeds in three parts. First, we highlight the importance and urgency of affording greater priority to an urban FP agenda by presenting an overview of the pace and scale of urban growth in Africa and Asia, noting its attendant challenges in terms of the growth of slums and inequality. Second, we summarize the state of knowledge on urban FP, focusing on research addressing low-income urban groups and settings of sub-Saharan Africa and South Asia. The broad methodological and geographical dimensions of the field are outlined before evidence on intra-urban differences, and issues that are of particular salience in the urban context, are described. An overview of this kind is overdue given that the last review on the topic was published nearly 30 years ago, when the majority of LMIC populations were rural (9). Third, we discuss the implications of these trends and bodies of evidence for future research.

## Background: Why Is Urban FP Important, and Why Now?

The importance of recognizing and implementing an *urban* FP agenda is highlighted by demographic trends of LMICs. At least four trends are noteworthy. First, the pace and scale of urban population growth are remarkable. The United Nations Population Division predicts that an additional 2.5 billion people will be added to the global urban population by 2050, with almost 90 per cent of this growth taking place in Asia and Africa (10). The same data indicate that by mid-century the urban population of Africa is likely to almost triple, while that of Asia is set to more than double. By that point, slightly over half of the global urban population will be Asian, with Africa's share exceeding one-fifth. While the contemporary pace of *urbanization* in these regions is not unprecedented in world history, the rates of *urban population growth* are higher than those observed for comparable periods in the developed world. Also unprecedented is the increase in the number of cities with very large populations of over one million, with most located in developing regions. Even so, the majority of future urban growth in LMICs will take place in smaller cities and towns that host higher rates of poverty and are the least equipped institutionally to deal with the challenges arising from rapid growth (10–12).

Second, this growth is associated with significant poverty and material deprivation in the form of slum-like urbanism. In sub-Saharan Africa, ~55 per cent of the urban population lives in slum-like conditions, compared with just over 30 per cent in the case of South Asia^1^. While slums are the focus of much research and practical literature dealing with urban poverty, the urban poor are not always concentrated in these areas—in India, for example, more than 80 per cent of poor urban households may in fact live in non-slum neighborhoods (11, 13). Moreover, we should note that a “slum” is sometimes an “informal settlement”, however these terms are not synonymous (14) and as such this paper uses the specific terminology employed in the research under review. Regardless of terminology, the wider issue of inadequate housing and service provision for rapidly growing urban populations is a dominant theme of the planning literature, although it is never explicitly framed in relation to issues of fertility or FP (15–17).

Third, and contrary to popular belief, over half of the expected growth in urban populations of LMICs is due to natural increase rather than rural-urban migration (3, 18, 19). Indeed, a series of studies have made the forceful argument that urbanization—both historical and contemporary—is better understood as a demographic rather than a purely economic process, particularly if we are to account for the phenomenon of “urbanization without economic growth” so often discussed for the African context (18–22).

Fourth, urban fertility rates have unexpectedly plateaued in many sub-Saharan African countries at levels well-above replacement and in a few cases have actually increased. A detailed analysis shows that fertility has stalled in about half of Africa's capital cities, at an average level of 3.4 births per woman, with recent increases in a few countries, including Nigeria, Democratic Republic of the Congo (DRC), and Tanzania. In other urban areas, stalls are apparent in about one-third of countries (23, 24).

The causes of African fertility stalls—which have been identified for contexts in different stages of development and the fertility transition, with variable patterns of urban and rural distribution—currently are not well-understood (25, 26). Researchers have variously pointed to contemporaneous trends in socioeconomic development (27), declining national and international support for FP programmes leading to greater unmet need and lower contraceptive use (28), high levels of desired fertility related to socioeconomic uncertainty (29, 30), as well as disruptions to female education linked to the effects of economic crises (and ostensibly structural adjustment programmes) of the 1980s and 1990s (31, 32). Regardless of their precise drivers, if fertility stalls continue then existing rates of urban growth will be sustained in future decades, potentially undermining the influence of urbanization in driving wider fertility and demographic transitions (23). Continued high fertility rates may have negative implications for short- and medium-term progress in achieving the SDGs in cities and towns, perpetuating higher rates of poverty while placing pressure on housing stocks and urban services ranging from clean water and sanitation to public transport.

Taken together, these trends demonstrate the need for health research and policy to take greater account of urban demographic changes in LMICs, and to afford greater priority to urban FP interventions in those regions. There is a limited but growing body of research that examines urban FP dynamics in LMICs from a range of disciplinary perspectives. As a result, there is a need for an overview of the evidence of how FP supply, demand, and programmatic interventions take specific forms, with various outcomes, in different urban contexts.

## Approach and Methods

A critical scoping review was undertaken of the urban FP literature to identify key topics of research interest as well as knowledge gaps or needs. While systematic reviews are widely applied in health and other disciplines, they are less useful when applied to a literature that is relatively small and comprised of diverse concepts, methodological approaches, and types of evidence. Scoping reviews are a more suitable approach when the purpose is to identify knowledge gaps, scope a body of literature, clarify concepts, identify the types of evidence available and used in a given field, and to examine how research is conducted on a particular topic (33). Critical reviews, for their part, move beyond the description of research findings to undertake additional analysis and critical reflection, usually with the aim of making a conceptual contribution to the literature (34, 35).

Applying a critical scoping approach, the objectives of this review are 4-fold. First, we identify the various methodological approaches and types of evidence employed in the study of urban FP in sub-Saharan Africa and South Asia. Secondly, we outline the geographies of this research interest at the regional, national, and urban scales. Thirdly, we describe the key findings of studies focusing on the intra-urban dynamics of FP. Finally, we present a set of themes and topics to inform future research in the field. The review process was guided by the question: *What literature exists on the topic of urban FP in sub-Saharan Africa and South Asia, what are its methodological and geographic dimensions, and what priorities for future research can be identified?*

The search strategy was iterative, constructed through the identification of relevant terms, concepts, and topics emerging during the search process. We searched Web of Science and Google Scholar online databases for English-language articles and reports published since the year 2000 with combinations of the terms “family planning,” “contraception,” “urban,” “rural-urban,” “urban-rural,” “intra-urban,” “migration,” “slum,” “informal,” “neighborhood,” “development,” and “fertility.” Gray literature not published in academic books and journals was included in the search. Identified works were mined for further references. Moreover, several key experts in the field were approached and asked for recommendations of significant recent studies and research focus areas that should be included. The search process took place between 1 May 2019 and 1 May 2020.

The criteria for including studies were as follows. First, they should be empirical research outputs (excluding descriptions of study protocols) with FP as a principal focus, or including a sustained engagement with FP issues within a wider health lens. Research only tangentially addressing FP as a minor aspect of more general health services and dynamics was excluded. Second, included works should focus on urban areas or communities, or show a sustained engagement with urban dynamics as they relate to those of rural areas. Those addressing “semi-urban” areas were excluded. Third, the research should address contexts of sub-Saharan Africa or South Asia, or examine such contexts within a larger LMIC ambit. Finally, we only included works that were published or released in 2000 or thereafter. Studies were selected by initially screening titles and abstracts to exclude irrelevant works; the full text of remaining studies were then assessed for eligibility. A total of 279 studies were deemed to have met all criteria for inclusion.

In addition, we employ Demographic and Health Survey (DHS) programme data on fertility, unmet need, and contraceptive use. These were accessed via the STATcompiler platform^2^.

## Analysis

The 279 studies included in the analysis were categorized according to their predominant source of evidence and geographical focus (Table 1). Source of evidence groupings included surveys, interviews/focus groups, mixed methods, and ethnography. Those employing surveys were further broken down according to the specific use or design of those methods. A significant majority of studies rely solely on survey evidence. Of these survey-based studies, over 60 per cent are cross-sectional in design, employing either Demographic and Health Survey (DHS) programme data or other survey sources, while longitudinal studies comprise little under 15 per cent. Nearly one-fifth of all survey-based studies rely on data from one or more DHS rounds, which only provide information on national urban-rural differences or aggregate trends in capital cities. While ethnography is sometimes conducted in combination with other methods, only one study relied exclusively on ethnographic analysis.

**Table 1:** Number and proportion of reviewed studies, by category.

**Variable**	**Number of studies** **(*n* = 279)**	**Proportion of total** **(%)**
**Methodology**		
Survey	225	80.6
Interviews/focus groups	30	10.8
Mixed methods	23	8.2
**Survey design**		
Other cross-sectional	114	40.9
Longitudinal	33	11.8
DHS cross-sectional	24	8.6
DHS multi-round	19	6.8
Multiple survey types	10	3.6
Quasi-experimental	10	3.6
Other multi-round	9	3.2
Randomized trial	4	1.4
**Geographical region**		
West Africa	80	28.7
East Africa	73	26.2
South Asia	51	18.3
Southern Africa	35	12.5
Multiple regions	31	11.1
Central Africa	7	2.5
**Urban focus**		
Large city	83	29.7
Multiple cities	70	25.1
National urban-rural	58	20.8
Urban/rural sites	20	7.2
Subnational urban-rural	18	6.5
Secondary city	14	5.0
National urban	12	4.3
Town	3	1.1
**Specific urban focus**		
Slum/informal settlement	23	8.2
Urban poor/non-poor comparison	23	8.2
Slum/non-slum comparison	19	6.8
Urban poor	8	2.9
Peri-urban	7	2.5

Studies were then grouped according to their country and region of focus—individual countries were counted as a focus only if they formed part of a study addressing five or fewer city or country contexts. African regions were defined according to the categories employed by the African Development Bank^3^. Table 1 demonstrates the prominence afforded to urban FP contexts and dynamics in West and East Africa, with those of South Asia as a lesser priority.

**Figure 1:**
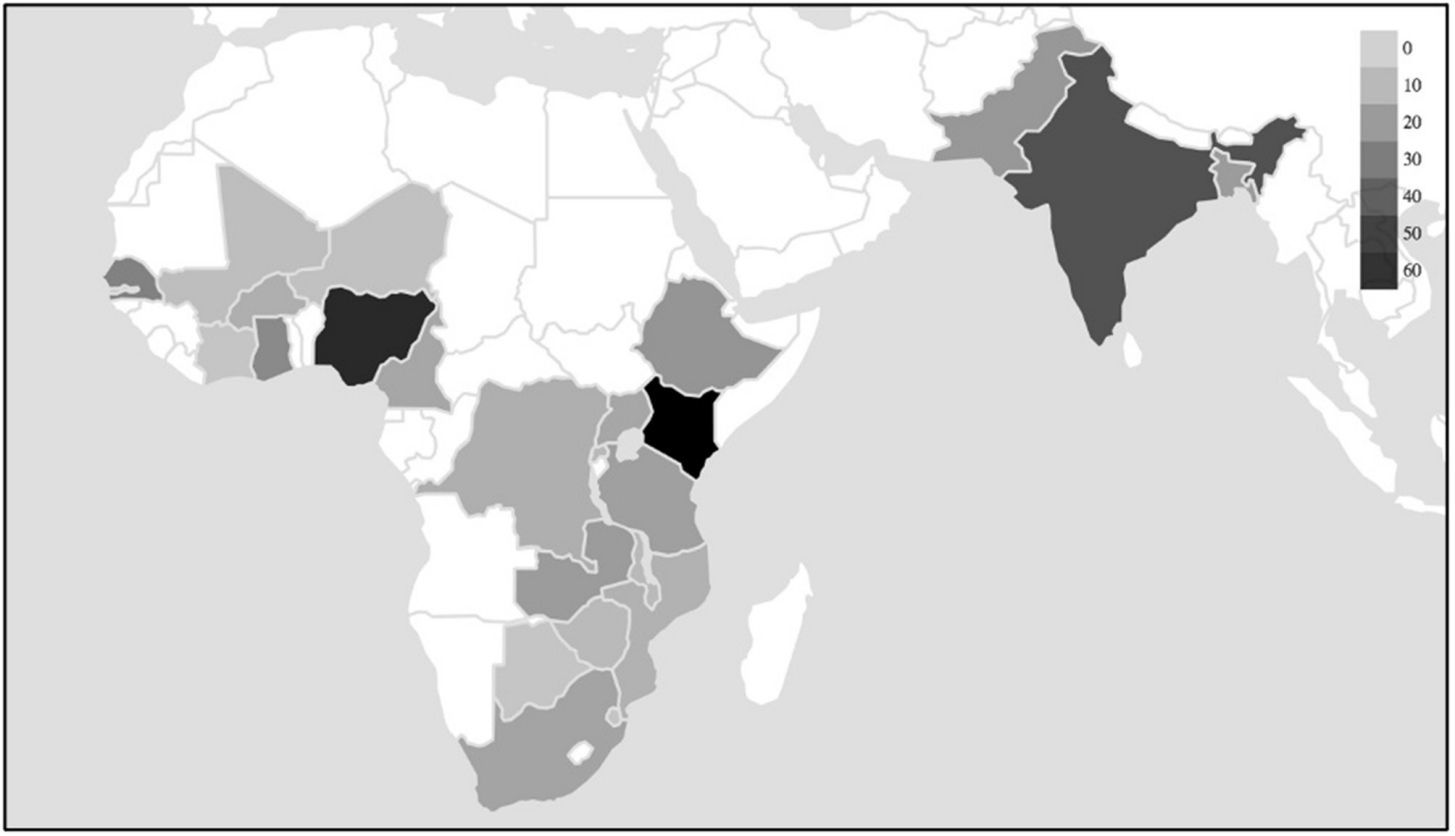
Number of studies, by country.

**Figure 2:**
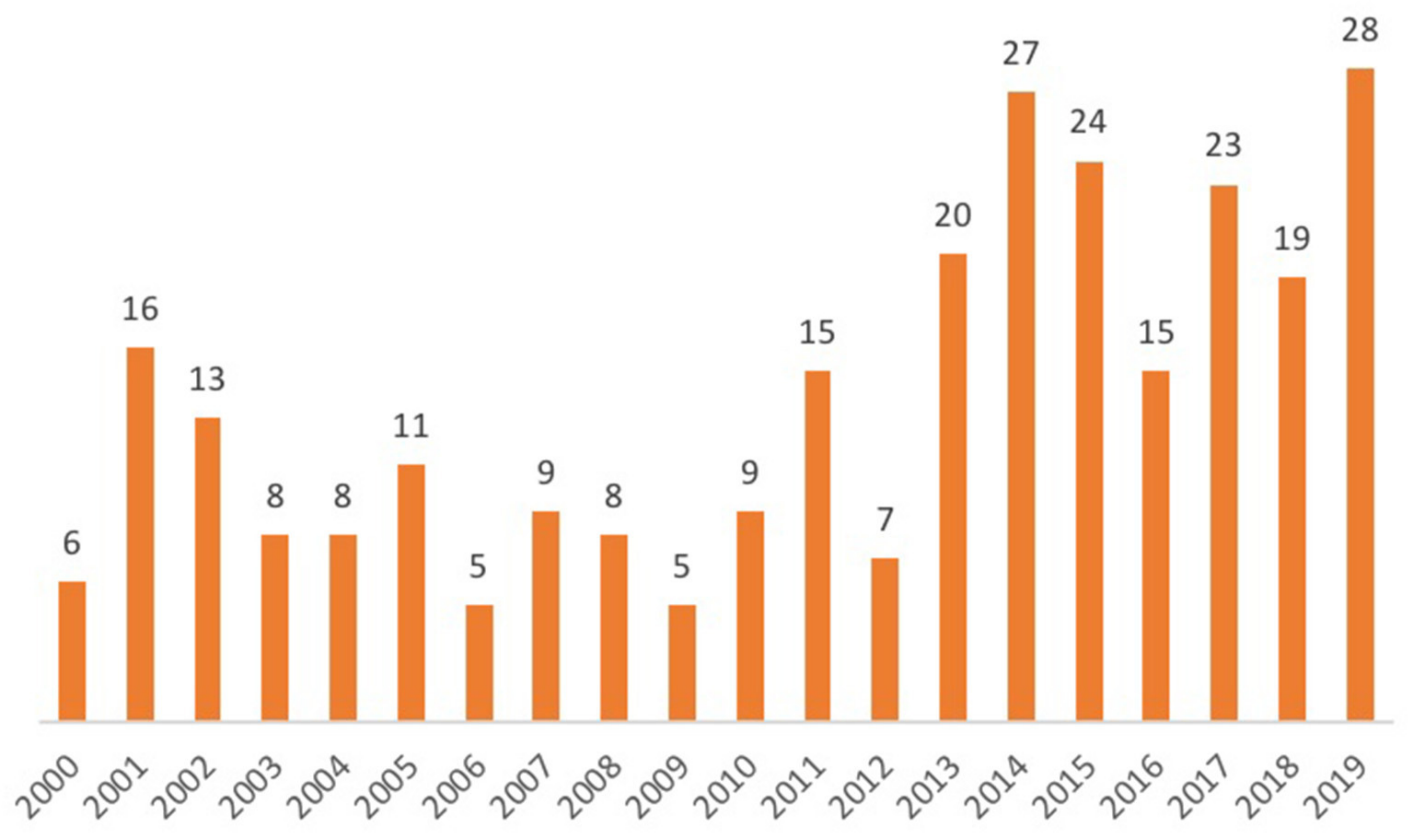
Number of studies, by year.

The priority given to African settings within the wider research field shows continuity with the region's emergence, since the 1990s, as the “new frontier” of FP (36): a frontier characterized by challenges related to high fertility rates, the HIV/AIDS epidemic, poor economic performance, the lowest prevalence of contraceptive use of any world region, and high rates of adolescent pregnancy. The geographies of research interest identified by this review reflect the outcomes of recent large-scale programmes such as the Urban Reproduction and Health Initiative (URHI), which implemented interventions and monitoring activities in Nigeria, Senegal, Kenya, and India, resulting in significant publication output from those countries (Figure 1). The influence of donor-supported programmes is also demonstrated by the increase in the number of studies produced annually over the past decade; this is represented in Figure 2.

The ways in which the FP literature addresses urban spaces and dynamics varies, as revealed in Table 1. Large cities with populations of over one million are the predominant focus of this work, although it has become increasingly common for researchers (as with those linked to the URHI programmes) to employ multi-city methodological designs that may include both large and secondary urban areas. However, overall smaller cities and towns remain a lesser focus. Large-scale surveys such as the DHS produce nationally representative data and are often employed for the purpose of urban-rural comparisons—this was the approach taken by over one-fifth of all reviewed studies.

Only around 30 per cent of all included studies addressed intra-urban realities and differences. As shown in Table 1, the dominant approach has been to examine spatial inequalities (in the form of a focus on slums or informal settlements, or a comparison of slum and non-slum areas) and economic inequalities (in the form of a focus on poor urban populations, or a comparison of poorer and wealthier income groups). Only seven studies included a specific focus on peri-urban areas, although it should be noted that many slums or informal settlements are likely to be located on urban peripheries without this being specifically noted.

Studies were grouped according to broad thematic headings although, given the diversity of methodological approaches and empirical foci existing within the field, thematic analysis was not prioritized. Close to three quarters of all reviewed works examine issues related to FP demand and use. Thirty-two focus exclusively on supply-side issues, marginally fewer than those addressing a combination of supply-side and demand-side factors. Around 40 per cent of all works examine issues related to (or determinants of) FP use and outcomes, while approximately one-fifth provide more descriptive analyses of FP knowledge, attitudes, and practices. In lieu of a detailed thematic analysis, the following section summarizes the emerging evidence on key FP topics, highlighting what we know about inter-urban and intra-urban differences.

## What do we Know About FP in Urban Areas?

### Unmet Need

**Figure 3:**
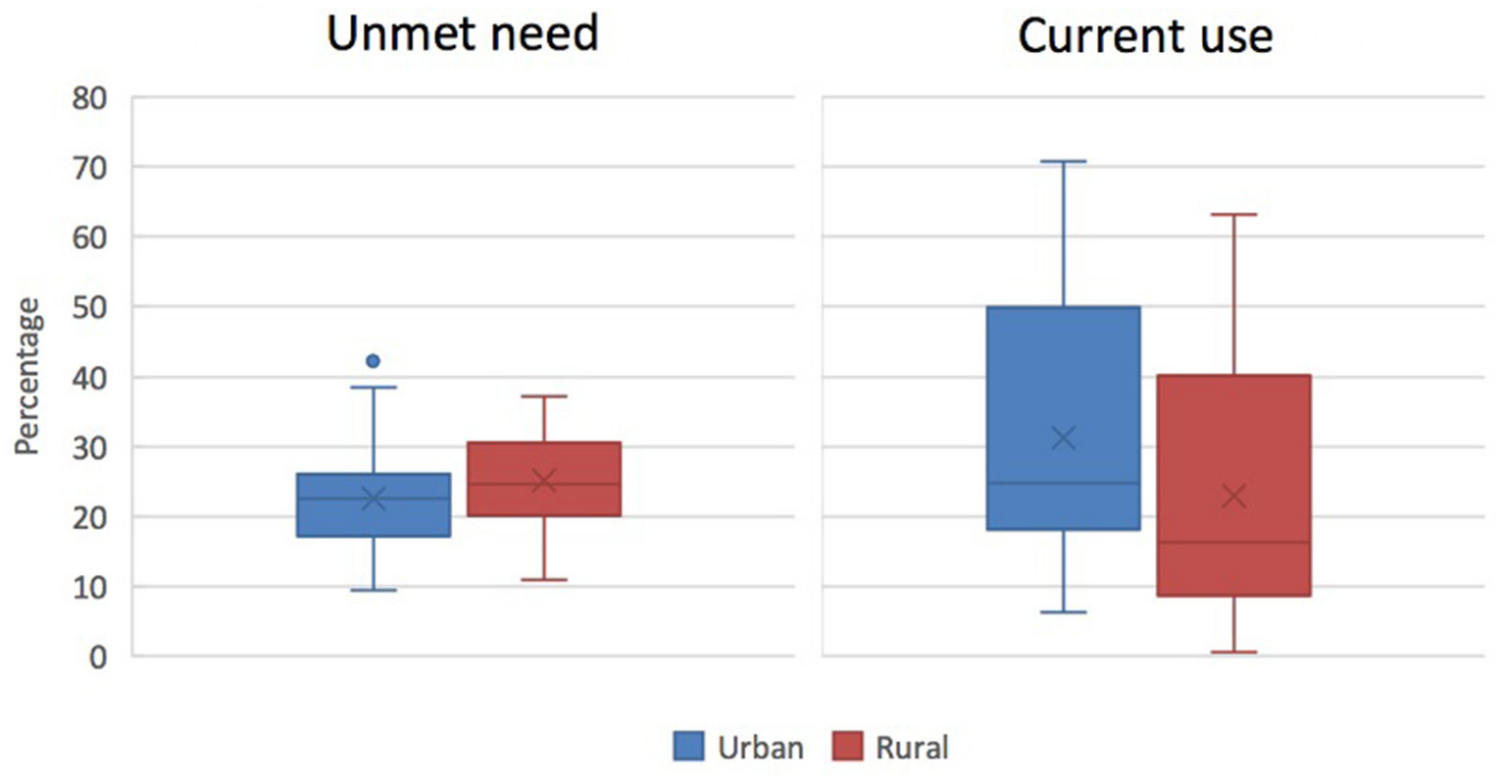
Percentage of currently married or in-union women with an unmet need for FP (left) or currently using any modern method of contraception (right) in sub-Saharan Africa, according to place of residence (source: ICF, 2015. The DHS Programme STATcompiler. http://www.statcompiler.com. Accessed May 27 2020).

Generally, both the desire to limit family size and contraceptive use are higher in urban than rural areas. That said, unmet need for FP, defined as non-use of contraception among women wishing to limit or avoid pregnancy for at least the next 2 years, is often similar between the urban and rural sectors and, in some settings, urban levels are higher than rural. Figure 3 shows the broad urban-rural comparison for sub-Saharan Africa. In 10 out of 39 African countries with relevant data, unmet need is higher in urban areas than in rural areas^4^. In South Asia, unmet need is generally higher for rural populations but only marginally so, as in the case of India (12.1 per cent urban vs. 13.2 per cent rural)^5^. More detailed spatial analyses reveal that patterns of unmet need in a country such as Ghana show significant geographic heterogeneity, including variations between particular urban communities, suggesting that bridging inequality gaps in contraceptive use calls for area-specific programmes (37).

Large differences in unmet need and unwanted childbearing exist according to wealth status in urban areas (38, 39). In Kenya, for example, while the Nairobi region enjoys the lowest levels of unmet need nationally, in slum areas within Nairobi the proportion of women with an unmet need was double that of the city as a whole and higher than that of rural women (40). While a greater proportion of poor urban women might use contraception relative to rural women, unmet need can still be higher for the former group.

Aggregate urban trends mask inter- and intra-urban differentials in fertility and FP outcomes in sub-Saharan African contexts. For example, we know that poor urban youth are more likely to engage in high-risk sexual activity (41–46). Moreover, poor young urban women are at particular risk of experiencing unintended pregnancy (47). Research has shown that the determinants of unintended pregnancy may vary by city and by settlement type (48). For women living in slum areas of Nairobi, for example, age, parity and marital status each had a significant net effect on the likelihood of unplanned conception, while marital status and ethnicity were more significant determinants for those residing in less deprived areas of the city (49). One longitudinal study showed that women in one slum neighborhood of Nairobi were almost twice as likely to realize baseline fertility preferences than those living in a different slum area (50).

High levels of unmet need and unintended pregnancy reflect constrained access, broadly defined, to contraceptive services, though an additional influence is that the childbearing preferences for urban women, as well as rural women, may be ambivalent and changeable (51).

The urban dynamics of unmet need, unstable fertility intentions, and unintended pregnancy may be related to an increasing resort to induced abortion in urban areas. Lifetime abortion rates are remarkably high in many LMIC urban settings (11). In Yaoundé (Cameroon), over one-fifth of young women reported having had an abortion; over 8 per cent reported more than one (52). In some cases—particularly contexts with restrictive abortion laws—many procedures are performed under unsafe conditions and result in complications (53–56). In Malawi, for example, a significant proportion of poor urban women procure abortions from traditional healers (39 per cent) or perform it themselves (14 per cent). For this group, an estimated 55 per cent of procedures result in complications with 17 per cent of these going untreated (57).

### Family Planning Supply

As mentioned above, high levels of unmet need in urban areas suggest the existence of supply-side barriers to FP commodities and services, among other kinds of obstacles. For example, weak governance systems affect FP supply in African urban areas due to fractured and confusing divisions of responsibility. These realities are true not just for reproductive health but all areas of urban management. In some cases, uncertainty over which part of government is responsible (legally, financially, or operationally) for FP provision has resulted from well-intentioned devolution processes that aspired to give more power to the local scale and to larger cities in particular. In Kenya, for example, the devolution of health services over the past decade saw the national government retain strategic oversight with subnational county governments tasked with decision-making and the delivery of services, including FP. The resulting mismatch of strategic capacity and local delivery mechanisms has resulted in national policies and guidelines that are less attuned to local contextual factors and needs; government ownership has been undermined and processes of funding, procurement, and monitoring complicated (58–60).

There may be limited overall penetration of public-sector FP services into LMIC cities, but especially into smaller towns and deprived areas and slums. This may be the case for various reasons. Smaller cities are not always seen as urban centers or are poorly connected into national supply and service chains. Even in larger cities, slum populations and settlements may lack legal recognition from public authorities and are thus neglected by formal service provision (38). In addition, the perceived dangers of working in such areas may increase absenteeism of health and FP staff (61). As such, poor urban populations are often dependent on private sources that may offer services of low quality and high cost. This implies that the urban poor, particularly residents of slums, are likely to be most affected by disruptions to the public provision of FP services (38).

The importance of the private sector supply of FP in urban areas is demonstrated by recent research. Vulnerable and harder-to-reach groups in Kenya and Nigeria, for example, are more likely than other urban women to obtain their short-acting methods from drug stores or pharmacies (62). In Bangladesh, researchers found a trend toward greater use of private sources in poorer urban areas (63). While a previous study found little evidence to suggest that expansion in private sector supply is contributing to inequalities in the level of contraceptive use in urban areas (64), it is possible that in some contexts this expansion is associated with the generation of spatial inequalities in the supply environment. In Nigeria, for instance, urban areas served by a good public supply environment enjoy an increased likelihood of having a better private sector supply, suggesting that neither sector acts to address the other's supply shortfalls (65).

We know from Kenya that where urban women go to obtain contraceptive methods is influenced by issues of service quality, cost, distance, and the specific kinds of service offered. Some research suggests that Kenyan women seeking FP services are less likely to use public sector facilities (despite the lower cost and more expansive offering of methods at those facilities), preferring private and other non-public facilities for reasons including higher perceived quality, shorter waiting times, and better client-provider interactions (66, 67). Private sources may be attractive to many poor urban women for various reasons, but public facilities, including hospitals, nonetheless remain important sources of better quality, affordable, and professionally-administered FP services (66, 68). In some cases, service quality has been shown to exert a greater influence on FP practices than geographic access. Urban supply environments in Senegal, for instance, are already dense; increasing the number of facilities or the range of services offered at facilities are not likely to increase FP use (69). Such findings suggest that urban women often bypass their nearest facilities for reasons of quality.

Provider restrictions present another set of supply-side barriers to FP access in urban settings. Restrictions based on age, marital status, parity, and spousal consent have been identified in urban settings of sub-Saharan and South Asia (70–73). Urban women at greatest risk of unwanted pregnancy often face barriers in obtaining more popular contraceptive methods including the pill, condoms, and injectables (73, 74). In some cases, they are more likely to face restrictions from private providers than from other provider types—a problem given that significant proportions of urban populations rely on private sources (72, 74, 75).

### Contraceptive Use

The use of modern contraceptives in LMICs is generally higher for urban than rural women. Figure 3 represents this difference for sub-Saharan African countries. Urban-rural differentials in current use are often small in countries with higher overall contraceptive prevalence (76). For example, no significant geographical differences in modern contraceptive use can be observed in countries such as Malawi (61.4 per cent urban vs. 57.5 per cent rural), South Africa (54.6 per cent urban vs. 52.5 per cent rural), and Bangladesh (56.2 per cent urban vs. 53.2 per cent rural)^6^.

Patterns of contraceptive use may vary significantly between population groups, regions, and cities within countries (77). Within cities themselves, women living in slum areas tend to report lower levels of use (78), although contexts such as India, Bangladesh, and Kenya (countries that have introduced concerted national FP programmes) have seen rapid increases in the use of FP by slum residents (39, 63).

With respect to method use, LMICs show markedly different contraceptive method mix profiles and preferences. It is therefore difficult to make any broad statements about urban and rural trends and differences (79). Trends away from the use of longer-term methods by urban populations have been observed for some South Asian and sub-Saharan countries. In urban areas of Pakistan, for example, the method mix has shifted away from sterilization toward an increased use of condoms (80). In Kenya, the trend has been for urban women to increase their use of short-term, often less effective, methods, with the proportion of women using long-acting methods (such as intrauterine devices and implants) dropping considerably in some cases (39, 81). Given that rates of discontinuation and failure are higher for short-term methods than for intrauterine devices or implants, a growing reliance on these methods may explain why urban areas of Kenya see high rates of unplanned pregnancies and births despite relatively high levels of contraceptive use.

In both African and Asian settings, there may be a general trend for more educated and wealthier urban residents to rely on traditional methods as part of a wider strategy of fertility regulation that can encompass occasional resorts to emergency contraception and safe abortion (82, 83). This may be linked to previous experiences of side effects from hormonal methods leading to method dissatisfaction (84). Ghana is a particular case in point of these trends, and has been extensively studied (85–89). Very high levels of traditional method use have also been documented in Ouagadougou (Burkina Faso), Lagos (Nigeria), and Kinshasa (DRC) (90–92). These kinds of ideas and practices can be expected to become more significant and widespread as urbanization proceeds and the middle-class grows in LMIC contexts, but presently it is unclear whether or how such attitudes and practices are diffusing among poorer urban women.

### Method Satisfaction

Experience of side effects from hormonal methods leading to method-related dissatisfaction is commonly reported as a reason for the discontinuation of contraceptive use among urban women (85, 86, 93–95). DHS data shows that urban women in low and middle-income countries are more likely to cite “health concerns” as a reason for non-use of contraceptives than rural women (96). The role of health concerns in giving rise to method dissatisfaction can vary between urban and rural contexts and may be specific to certain methods. In Kenya, for example, perceptions of safety surrounding the long-term use of injectables were found to have a significant effect on method satisfaction in the urban study site—a slum area of Nairobi—but not in the rural site (84).

### Discontinuation and Switching

Generally, sub-Saharan African populations show high rates of contraceptive discontinuation and low probabilities of switching between methods, indicating high risks of unintended conception (97). Ambivalent, variable, or low motivations surrounding childbearing may be associated with discontinuation among urban African women, particularly if they experience method-related dissatisfaction (50, 51, 98).

Poor urban women experience high rates of contraceptive discontinuation (47). Among women living in slum areas of Nairobi (Kenya), almost half of all women who adopted a method during the postpartum period discontinued (primarily due to method-related dissatisfaction) within 1 year, a proportion far higher than the national estimate of 32 percent (94). However, the same women were also far more likely than the national average to switch to another method within 3 months of discontinuing, possibly reflecting a high degree of motivation to postpone pregnancy among couples living in slum areas. By contrast, urban women in Uttar Pradesh (India) who use multiple contraceptive methods were found to experience higher incidences of pregnancy and abortion, suggesting problems with switching and lapses in coverage between methods (99). From research in Senegal it is also evident that the likelihood of having an unmet need for contraception after discontinuation can vary significantly between particular urban contexts (100).

### Determinants of and Barriers to Contraceptive Use

As mentioned, an emphasis of many urban FP studies has been understanding the specific factors and mechanisms that promote or inhibit contraceptive use. Research in this area has seen considerable progress toward the use of more sophisticated theoretical and methodological approaches. Two decades ago Casterline and Sinding (101) criticized mainstream research on unmet need for “simplistic theorizing” in the absence of sound models of behavior that consider how fertility *preferences* may operate under various *constraints*, and how they compete with other preferences. By contrast, some researchers now employ models of behavioral change such as the “theory of planned behavior” (102–104) and “theory of reasoned action” (105), emphasizing the study of intentions as a predictor of human behavior alongside the factors that inhibit the translation of intention into behavior. These may include personal factors (such as knowledge, skills, or self-efficacy) as well as wider social norms (106). Further, in recent scholarship greater emphasis has been placed on the role of communication in shifting urban FP intentions and behaviors (107).

Studies of the determinants of modern contraceptive use have often focused on the effects of individual-level factors (such as age, education, and parity) on the probability of modern FP use. Fewer have assessed how FP use is associated with household or community-level factors. However, there has been an increase in studies examining “contextual effects on health behaviors” (108). Accordingly, recent work explores how exposure to FP messages, interpersonal communication, perceived social risk, religious factors, ethnicity, and poverty affect reproductive intentions and behaviors among urban groups. For example, several studies show that partner or spousal discussion plays an important role as a mediating factor between exposure to FP messaging and contraceptive use in African cities (104, 109).

The study of social norms has long been an interest of health and FP researchers. However, there is evidence of growing emphasis on the role of social norms in influencing fertility behaviors at individual and collective levels, in addition to their effects on the supply of services (110, 111). Sex composition of surviving children and desire for male children have been shown to be important determinants of fertility behavior, contraceptive use, and method choice in urban slum areas of South Asia (112–114). In some Nigerian contexts, son preference is also associated with negative attitudes toward FP among poor urban men (or the perception of such attitudes held by their partners) (115). Pronatalist norms have been shown to retain important influence over FP behaviors in urban areas of Niger (102, 103). It is not clear how the nature or influence of such norms are changing with the progress of urbanization (with resulting changes to family structures, lifestyles, and cultural influences) but several authors have argued that this is happening in particular African and Asian cases (103, 116, 117).

There have been recent attempts (linked to the URHI country programmes) to examine the specific mechanisms through which normative factors influence FP opinions and behaviors, and in particular the role that communication plays in mediating the relationship between social norms and contraceptive use (107, 109, 110, 118). In one study researchers draw on the “theory of normative social behavior” alongside a mixed-methods approach to assess precisely the role played by injunctive norms (social sanctions for failure to conform) and interpersonal discussion (between spouses and others) in influencing the relation between perceptions of others' behavior (descriptive norms) and contraceptive use among the urban poor in India (110). They found that spousal influence and communication were indeed important determining factors in decision-making, noting that norms surrounding contraceptive use are changing rapidly in the context of urbanization and expanding access to health information. However, precisely how different types of norms influence specific kinds of decision-making behavior in particular urban contexts remains poorly understood (110).

### Programme Interventions

Well-designed multi-component FP interventions, encompassing both supply-side and demand-side activities, can make a significant difference to contraceptive use among urban populations. Recent evaluation studies of programmes such as the Urban Reproduction and Health Initiative (URHI) and The Challenge Initiative (TCI) provide useful insights for urban programmatic planning. Positive effects on FP use linked to project interventions were observed for all country programmes (119–122). For example, the Kenyan programme helped to boost the proportion of urban women using modern contraceptives from 45 to 52 per cent in the 4-year follow-up period (122). In Nigeria, that proportion increased from 21 to 31 per cent in the same period (120).

Studies such as these provide an informed basis to assess the impact of supply- and demand-side interventions on increased contraceptive use, particularly among the urban poor. Considering only the impact of supply-side interventions focused on increasing service availability and quality, a longitudinal and comparative assessment of the African URHI country programmes found that numbers of new acceptors at targeted facilities rose in all three contexts, while the overall number of clients increased in Nigeria and Kenya (123). The success of various supply-side interventions differed according to regional or national context—issuing information and educational materials at facilities increased users in Nigeria, but not in Kenya or Senegal, while enhanced provider training had a significant positive association with user numbers in Nigeria and Kenya.

For demand-generating activities specifically, in all URHI contexts outreach activities (implemented by FP or community health workers and local radio programming) were significantly and positively associated with increased modern method use by midterm (124). Television programmes had a significant effect on use in India and Nigeria, while exposure to project-related messaging and logos through various media were also significantly associated with improved use in Kenya and Nigeria (120–122). Results from Kenya indicate that demand-generating activities such as radio programming can be the most cost-effective means of promoting increased use of modern methods (122). FP outreach through mobile service delivery has been shown to be effective in increasing the use of FP services in four cities of Nigeria (107). These kinds of measures take on particular importance in poor urban contexts where security concerns, a lack of appropriate infrastructure, and high levels of built environment density may inhibit government service delivery efforts.

While such evidence demonstrates the effectiveness of targeted and multi-level interventions in increasing the use of contraceptives in urban areas, including among poor populations, their positive effects may not always be sustained beyond project completion. This highlights the need for programmers to consider longer-term strategies that would support the continued effects of project components following their implementation (125).

Some reproductive health programmes have specifically targeted the integration of FP with other healthcare services. In principle, integration offers an economy of service provision and is therefore valuable in contexts with limited resources. Recent studies provide evidence of the impact of such programmes. Evaluations of the Senegalese URHI programme suggest that the integration of FP services with those of maternal and child health presents a significant opportunity to reduce unintended pregnancies in urban areas (126). The equivalent Kenyan programme revealed that integration-related interventions had mixed results across different service areas, highlighting time and workload constraints on the part of providers as barriers to effective integration (127). Indeed, the South African experience shows that personnel and facility-level issues may be compounded by wider political and policy uncertainties and the problems of generally overburdened public health systems (128). These challenges point to the need for flexible partnership-based approaches to service integration involving communities, healthcare providers, and other actors (129). Based on randomized controlled trial evidence from Mumbai (India), partnership models (involving community resource centers operated by non-governmental organizations) for the delivery of integrated health services have been found to be a feasible and potentially replicable approach for promoting health in urban informal settlements (130, 131).

The overall implication of this research is that *context matters* in significant ways when designing FP and related healthcare programmes. Cities and towns—even those within the same country—all have unique social, economic, and political characteristics that affect how services should be delivered, and how those services will be taken up and used. While this conclusion may seem disabling in the search for universal lessons and recommendations, the significance of context highlights at least two points. First, it affirms the importance placed on appropriate monitoring and evaluation mechanisms to accompany FP interventions and thereby inform evidence-based planning and mid-term programmatic adjustments according to local conditions, as emphasized by recent initiatives such as URHI and TCI. Second, it calls for further research that moves past broad urban-rural binaries and national comparisons to examine the specific dynamics that unfold within and between various urban settings, as we discuss in the following section.

## What are the Implications for Future Research?

Enhanced FP interventions for the urban poor would bring considerable benefits for poverty reduction, maternal and child health, education, women's employment, and food security at multiple scales (38, 132–135). Some researchers argue that FP programmes—alongside industrialization or improved urban infrastructure and institutions—may be effective in slowing urbanization in poor countries and reducing the total share of their urban populations living in informal settlements (136). FP is thus a key strategy that governments can implement to support achievement of the SDGs (1).

However, to deliver on the potential of an urban FP agenda, we have some way to go to understand the precise nature of the challenges involved in improving the delivery and use of contraceptive services in different urban contexts. Here we discuss four broad priority areas for future research in the urban FP domain.

### Neighborhoods and Poverty

While the concept of “neighborhood” has long been an important basis of urban research in the global North, particularly North America, it plays a limited role in discussions of urban demography and FP in LMIC contexts (3). Important exceptions include the longitudinal research emerging from the few cities that host health and demographic surveillance systems. This work demonstrates the importance of the neighborhood context in shaping reproductive ideas and behaviors (50, 84). Recent studies analyzing health inequalities in Accra (Ghana) by linking satellite imagery with survey and administrative data have also taken a specific interest in assessing neighborhood effects (8, 137–139).

There is a need for further research assessing the role of neighborhood effects (including factors of social cohesion, social capital, collective efficacy, and community resources) in influencing sexual, reproductive health, and fertility behaviors and outcomes (3, 61). Researchers have made a start by describing “slum residence effects” for some LMIC settings (41–43). However, it should be recognized that not all “slums” are the same, and official definitions, material conditions, and socioeconomic characteristics of slum and informal settlements may vary substantially between urban and country contexts, and even within the same city (140).

In developing a more fine-grained understanding of the role of locality and place in affecting urban demographic change, the insights of sophisticated analyses of urban poverty and inequality should be retained. A key theme in this work is the analysis of heterogeneity, understood in both spatial and temporal dimensions (141–144). Among other things, the notion of spatial heterogeneity alerts us to the implications of particular slum-like conditions for FP service delivery and fertility patterns. This would include, for example, how the conditions of an older centrally-located settlement differ from those of a newly-formed community on the urban periphery in terms of access to healthcare, transport, and infrastructural services of all kinds. Moreover, the concept of heterogeneity highlights the diversity of experiences among the urban workforce and poor, including the specific kinds of barriers to reproductive healthcare faced by those working in the informal sector.

### Governance

Where and precisely how should FP be inserted or strengthened within the complex array of urban actors and institutions encompassing public, private for-profit, private non-profit, sectoral, municipal, state/provincial, and national governments? Are there examples of properly “joined-up” (11, 141) approaches to the governance of urban FP? Can such approaches deal adequately with rapid change in the spatial dimensions of urban growth and peri-urbanization in LMICs? In what circumstances are place-based strategies (with a slum focus) or people-based strategies (with a focus on the urban poor wherever they may live) more appropriate and effective? Answering these and similar questions of governance remains a core challenge, while the urgency of doing so is underscored by ongoing reforms driven under the banner of decentralization and devolution (3, 145). It is important that lessons from countries that have devolved health and FP services, such as Kenya (58, 60), are captured and shared across different contexts undergoing similar reforms.

There is now a need for further empirical research that examines the precise operations of the governance system—extending across multiple sectors and scales—that shape the dynamics of FP supply and demand in towns and cities. While examining mechanisms of (and barriers to) improving FP supply organization and service delivery within the health sector is important and necessary (146–148), it is not enough by itself. It is imperative to improve understanding of how FP services fit in the wider network of urban services and infrastructures (such as housing, water/sanitation, or public transport) and the ways in which these are designed, funded, and maintained. Here FP researchers may benefit by engaging with work emerging from other urban disciplines on the “hybrid” and multilevel governance systems that complicate urban management and development in LMICs (149–152). Without a grounding in the processes and difficulties of managing cities, FP and reproductive health professionals will be unlikely to forge the effective partnerships with the urban sector on which innovation in service delivery will depend.

Grasping the “actually existing” urban governance relations, processes, and practices that shape the delivery of and access to FP (as opposed to a preoccupation with formal health institutions and structures) would have at least two additional benefits. It would not only enhance our understanding of the drivers, barriers, and outcomes of contraceptive use, but also inform wider governance arrangements linking FP to the management of urban change. In particular, this kind of knowledge would be well-suited to feed into the development of what should be given priority within national urban policies. These policies are and will be key to delivering on the new urban development agenda by calibrating the powers and responsibilities held across levels and sectors of governance. However, to date they have been weak on health and demographic issues (153, 154).

### Migration and Displacement

While the majority of urban growth in LMICs is contributed by natural increase, migration remains a key trend and driver of that growth. Most research confirms that rural-urban migration has a downward effect on fertility rates, alongside positive effects on contraceptive use, indicating that migrants adapt to their new urban conditions and assume behaviors that are prevalent among permanent or more established populations (3, 155–158). Recent work has explored the precise influence of selectivity, adaptation, and disruption effects in particular contexts, noting that their relative influence may differ according to prevailing social, political, and economic realities (157, 159, 160).

Future research on FP and migration should take the diversity of migratory experiences into account. This means, first, recognizing that not all migrants are of similar age, that not all are poor and move into slum areas, that they may stay at their destination for differing lengths of time, and that some will move around urban areas more often than others during their lifetime. Second, the majority of migration studies focus on movements from rural areas into towns and cities, as such the literature is remarkably thin on how movements within and between particular urban areas affect reproductive behaviors and fertility (3, 161, 162). Third, there is a need for further work exploring how specific urban community contexts shape different migratory experiences, sexual transitions, FP needs, and fertility outcomes, particularly for large youthful migrant populations (163, 164).

At present we know little about the implications of increasingly significant urban migrant groups—including those displaced by civil unrest and disasters—for FP, including their particular service needs and the specific barriers to access they face (165–168). How to maintain FP and reproductive health services in disrupted environments will take on increasing urgency and a more urban character in the face of climatic, epidemiological, and political-economic threats and the “urbanization of displacement” (169–171).

### Resilience

A resilience perspective on health and demography shifts the emphasis from vulnerability (for example, of falling pregnant unintentionally) to the reasons why some urban groups are able to cope with and respond to adverse conditions (141). This, in turn, can provide guidance for positive action. Here the key question is how FP can form part of strategies to enhance the agency of poor urban residents and communities to be more resilient in the face of both long-term risks and shocks. These risks and shocks could include the urban manifestations and effects of climate change, food security, and infectious disease, for example.

Regardless of debates surrounding the potential role that FP should play within a global climate response (172–175), it should be recognized that fertility exerts a powerful influence on urban growth. As such, increasing access to FP is critical for easing the urban adaptation burden and enhancing resilience, particularly among more vulnerable populations (174, 176, 177). Doing so calls for further study of the associations between climate variability and reproductive goals and behaviors among urban groups, so that changing FP needs can be assessed and met (178).

Finally, considering the documented links between poverty, household food security, nutrition, and maternal and child health (135, 179), more research is needed to examine the urban links between FP, food systems, health, and fertility in a range of LMIC contexts. Enhancing our understanding of how FP interventions and fertility change affect nutrition and food security will be critical if we are to plan urban food systems and foster communities that are more resilient in the face of food price volatility, climatic stresses, extreme weather events, and a range of other external shocks (180).

## Conclusion

This review demonstrates that urban FP scholarship addressing LMIC settings is broad and varied in its specific objectives, methodological elements, and geographic and thematic foci. While limited in overall size, urban FP research output has increased significantly over the past decade as donor and government interest in urban population growth and urban health in LMICs has grown. However, key spatial and thematic gaps in our knowledge remain. FP research, as with urban health and development scholarship more generally, remains focused on large cities or broad urban-rural comparison, often overlooking transformations unfolding in smaller settlements and urban peripheries. We know too little about FP and demographic dynamics manifesting in different kinds of neighborhood and under various conditions of spatial and economic deprivation. Few studies examine or point to appropriate governance arrangements for FP in rapidly changing urban contexts. Diverse urban migratory pathways and experiences, with their implications for FP programming, remain understudied. So too do the precise roles of FP within strategies and processes to promote urban resilience.

We have argued for a closer link between the FP and urban sectors as a way to drive progress toward achieving the SDGs, and we have set out the initial parameters of a research agenda to inform that linkage. Practical steps are now required to move forward an urban FP agenda. Raising the profile of FP and its contribution to the long-term sustainability of urban areas and nations is the first essential step. This should be followed by engaging the commitment of urban leaders (such as city mayors), policymakers, planners, and other actors involved in multisectoral and multilevel urban reform. In drawing attention to this agenda, it should be emphasized that urban settings of LMICs, including locations within cities and towns, are diverse and rapidly changing. The development of FP strategies and priorities to address present and future needs must reflect those realities.

## Author Contributions

JD was involved in conceptualization, data curation, investigation, methodology, visualization, writing of the original draft, and reviewing and editing. JC, TH, MM, and IS was involved in conceptualization, writing of the original draft, and reviewing and editing. SP was involved in conceptualization, funding acquisition, supervision, writing of the original draft, and reviewing and editing. All authors contributed to the article and approved the submitted version.

## Funding

This work was supported, in whole or in part, by the Bill & Melinda Gates Foundation (INV-008737) through the IUSSP Family Planning, Fertility and Urban Development programme. Further support was provided by the PEAK Urban programme, funded by UKRI's Global Challenges Research Fund, Grant Ref: ES/P011055/1.

## Conflict of Interest

The authors declare that the research was conducted in the absence of any commercial or financial relationships that could be construed as a potential conflict of interest.

## Publisher's Note

All claims expressed in this article are solely those of the authors and do not necessarily represent those of their affiliated organizations, or those of the publisher, the editors and the reviewers. Any product that may be evaluated in this article, or claim that may be made by its manufacturer, is not guaranteed or endorsed by the publisher.

## Footnotes

^1^Data drawn from World Bank Open Data at https://data.worldbank.org, accessed 15 June 2020.

^2^https://www.statcompiler.com/en/, accessed 27 May 2020.

^3^See https://www.afdb.org/en/countries, accessed 17 July 2020.

^4^Data drawn from STATcompiler at http://www.statcompiler.com, accessed 11 June 2020.

^5^Data drawn from STATcompiler at http://www.statcompiler.com, accessed 26 May 2020.

^6^Data drawn from STATcompiler at http://www.statcompiler.com, accessed 11 June 2020.
